# Topographically Distinguished Microbiome Taxonomy and Stress-Response Genes of Royal Belum Rainforest and Raja Muda Musa Peat Swamp Revealed through Metagenomic Inquisition

**DOI:** 10.3390/ijms24010872

**Published:** 2023-01-03

**Authors:** Mohd Fadzli Ahmad, Hasdianty Abdullah, Muhammad Naim Hassan, Muhammad Imran Jamaludin, Ashvini Sivam, Kazuhiro Komatsu, Irni Suhayu Sapian, Halimah Alias, Mohd Noor Mat Isa, Victor S. Kuwahara, Nor Suhaila Yaacob

**Affiliations:** 1 Department of Science & Biotechnology, Faculty of Engineering & Life Sciences, Universiti Selangor, Bestari Jaya 45600, Selangor, Malaysia; 2 Institute of Bio-IT Selangor, Universiti Selangor, Jalan Zirkon A7/A, Seksyen 7, Shah Alam 40000, Selangor, Malaysia; 3 Faculty of Engineering, Shinshu University, 4-17-1, Wakasato, Nagano 390-8621, Japan; 4 Malaysia Genome and Vaccine Institute, National Institute of Biotechnology Malaysia, Jalan Bangi, Kajang 43000, Selangor, Malaysia; 5 Faculty of Education & Graduate School of Engineering, Soka University, 1-236 Tangi-Machi, Hachioji-Shi 192-8577, Japan; 6 Centre for Foundation and General Studies, Universiti Selangor, Jalan Zirkon A7/A, Seksyen 7, Shah Alam 40000, Selangor, Malaysia

**Keywords:** Raja Musa peat swamp, stress response, soil metagenomics, microbiome

## Abstract

Soil ecosystems are home to a diverse range of microorganisms, but they are only partially understood because no single-cell sequencing or whole-community sequencing provides a complete picture of these complex communities. Using one of such metagenomics approaches, we succeeded in monitoring the microbial diversity and stress-response gene in the soil samples. This study aims to test whether known differences in taxonomic diversity and composition are reflected in functional gene profiles by implementing whole gene sequencing (WGS) metagenomic analysis of geographically dispersed soils from two distinct pristine forests. The study was commenced by sequencing three rainforest soil samples and three peat swamp soil samples. Soil richness effects were assessed by exploring the changes in specific functional gene abundances to elucidate physiological constraints acting on different soil systems and identify variance in functional pathways relevant to soil biogeochemical cycling. Proteobacteria shows abundances of microbial diversity for 52.15% in Royal Belum Reserved Forest and 48.28% in Raja Musa; 177 out of 1,391,841 and 449 out of 3,586,577 protein coding represent acidic stress-response genes for Royal Belum and Raja Musa, respectively. Raja Musa indicates pH 2.5, which is extremely acidic. The analysis of the taxonomic community showed that Royal Belum soils are dominated by bacteria (98% in Sungai Kooi (SK), 98% in Sungai Papan (SP), and 98% in Sungai Ruok (SR), Archaea (0.9% in SK, 0.9% in SP, and 1% in SR), and the remaining were classed under Eukaryota and viruses. Likewise, the soils of Raja Muda Musa are also dominated by bacteria (95% in Raja Musa 1 (RM1), 98% in Raja Musa 2 (RM2), and 96% in Raja Musa 3 (RM3)), followed by Archaea (4% in RM1, 1% in RM2, and 3% in RM3), and the remaining were classed under Eukaryota and viruses. This study revealed that RBFR (Royal Belum Foresr Reserve) and RMFR (Raja Musa Forest Reserve) metagenomes contained abundant stress-related genes assigned to various stress-response pathways, many of which did not show any difference among samples from both sites. Our findings indicate that the structure and functional potential of the microbial community will be altered by future environmental potential as the first glimpse of both the taxonomic and functional composition of soil microbial communities.

## 1. Introduction

Peatland is an ecosystem that degrades organic matter from deteriorated plants generated through wet conditions. It covers approximately 4 million km^2^ and spans 180 countries worldwide. Southeast Asian peatlands constitute 6% of the world’s 24.8 million hectares of peatland [1]. Peat swamp forests are crucial to the global carbon (C) cycle and comprise one-third of soil carbon [2]. Tropical peatland is known as the peat swamp forest (PSF) in Malaysia, where flora and fauna survive in acidic and waterlogged waters, and the carbon balance is a key element [3]. It has a special ecological system. The North Selangor Peat Swamp Forest (NSPSF), the largest peat swamp forest complex on the west coast of Peninsular Malaysia, is located in the northwestern part of Selangor State. It covers an area of 73,392 hectares, comprising Raja Musa Forest Reserve (RMFR), with 23,486 hectares [4].

The Royal Belum State Park was initially known as Hutan Simpan Belum, Perak, Malaysia, in 1971, and eventually regazetted as a state park in 2007 and as the Crowning Glory of the Peninsula. The state park is considered one of the oldest, protected, undisturbed, and pristine landmasses in Peninsular Malaysia, being more than 130 million years old. It occupies 290,000 hectares between the Bang Lang National Park and Hala Bala Sanctuary in Thailand [5,6,7,8]. The forest is divided into two regions; the Upper Belum area, which expanded to the border between the country and Thailand, and the Temengor Lake area of Lower Belum [9]. It is claimed that the Royal Belum forest is more ancient than the Amazon and Congo forests [10,11]. Geographically, about 57% of its area is 80 to 300 m above sea level and 41% in the range 300 to 1533 m above sea level. The forest is classified as an environmentally sensitive area (ESA) rank 1 region, where growth, farming, and forestry are not permitted except for low impact tourism, study, and education. The Royal Belum forest is the habitat of Malaysia and the world’s largest and most significant natural ecosystems and habitats for in situ conservation of biodiversity [12].

Even though soil bacteria have been studied for more than a century, most soil bacteria remain undescribed in their diversity [13]. Soil microbial biomass competes for the soil mat biomass of plants or animals, with soil frequently comprising more than 1000 kg of microbial biomass carbon per hectare [14]. This is not surprising considering that soil bacteria are among the most abundant and diverse organisms on earth [15,16,17]. It is challenging to understand their basic contributions to ecosystem processes, including the cycling of nutrients and carbon, development of plants, stress-response effect, and greenhouse gas emissions [18,19]. Most soil bacteria do not fit those found in pre-existing 16S ribosomal RNA (rRNA) gene databases [20] due to scarce genomic details [20,21,22]. Furthermore, most soil bacteria have not been successfully grown in vitro [21,22]. Soils contain some of the most diverse microbiomes on Earth and are important in various aspects. It is vital to model the global pattern distribution and functional gene repertoire of soil microorganisms, together with environmental relations between the diversity and composition of soil populations [23,24], where biological mechanisms and functions resulting from the interactions among specific genotypes and their microenvironment are predicted to result in adjustments in soil structure beyond the size of individual cells [25].

The largest terrestrial carbon source is soil organic matter. The extent of the pool depends on the equilibrium between the production of soil organic matter from plant litter decomposition and mineralization into inorganic material [26]. Soil organic matter (SOM) is the essential instrumental agent that generates organizational complexity, where it functions to bind together mineral elements and colloids [25]. A severe loss of SOM content may degrade soil functionality, its capacity to provide essential ecosystem services, and soil health [27]. Soil pH, soil organic carbon (SOC), microbial biomass carbon (MBC), and available potassium (AK) are the main features significantly associated with the variation in soil fungal community [28,29]. Several studies revealed the microbial roles in SOM formation [30,31,32,33,34]. Nonetheless, Ref. [31] agree that the study demonstrating microbial residues account for the chemistry, stability, and abundance of SOM remains lacking. Previously, several studies have been revealed levels of organic matter such as nitrogen, phosphorus and carbon from Malaysia’s pristine forest soil and sludge. From previous studies, levels of organic matter from pristine forest soil are lesser than levels of organic matter from Malaysia’s and Japan’s sludge due to exposure of sludge to agricultural activities [35,36,37].

The microbial population is responsible for the synthesis of vital soil nutrients in a distinctive manner. The community must also interact with the heterogeneous soil environment, where resource bioavailability, osmotic and oxidative stress, temperature, and pH all impose restrictions on cell-specific functioning [38]. As a result, a high fraction of the genes in soil microbial genomes are involved in stress response, primarily environmental signaling, transcriptional factors, and protein chaperones [39,40,41]. This insight is critical for understanding how microbiomes respond to stressful environmental situations [42]. Soil pH is a strong parameter that determines the biodiversity patterns, especially for bacteria. This is due to either soil acidity or a confounding phenomenon in which soil pH acts as a proxy for a range of other parameters across soil environmental gradients. Acidic soils often have lower phylogenetic diversity and are particularly dominant with acidophilic and Acidobacteria lineage. The pH of most of virgin soils in Malaysia are acidic, such as Bera Lake Forest (4.24), Raja Musa (3.36), Ayer Hitam (5.81), Chini Lake (3.97), Langkawi Island (4.23), Kenyir Lake (4.83) [43,44,45,46].

The stress response governs many microbial functions, including starvation survival, antibiotic tolerance, antibiotic manufacturing, interactions with a eukaryotic symbiont, and atmospheric oxygen fixation. These functions aid the preservation of climax conditions at the ecosystem level. Here, we assessed whether known differences in taxonomic diversity and composition are reflected in functional gene profiles by implementing the whole gene sequencing (WGS) metagenomic analysis of geographically dispersed soils at two distinct types of pristine forest. Soil samples (three samples each) from the rainforest and peat swamp, previously collected as part of a Science and Technology Research Partnership for Sustainable Development (SATREPS) project, were sequenced. In addition to assessing the richness effects, we explored specific functional gene abundance changes to elucidate the physiological constraints on different soil systems and identify variance in functional pathways relevant to soil biogeochemical cycling.

## 2. Results

### 2.1. Taxonomic and Functional Comparisons of Tropical Rain Forest and Peat Swamp Soil Bacterium

**Figure 1:**
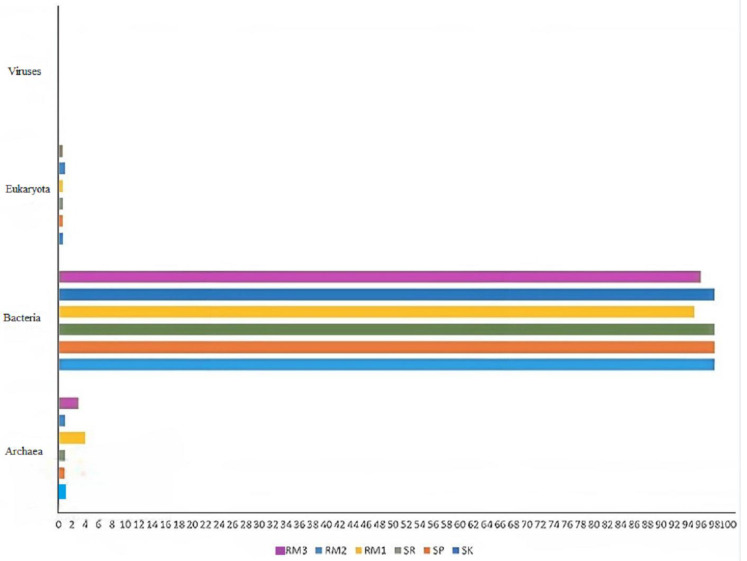
Microbial community domain in Royal Belum and Raja Musa soils generated with MGRAST.

Phylogenetic analysis was conducted using the Metagenomic Rapid Annotations using Subsystems Technology (MG-Rast) server, where metagenome data underwent Basic Local Alignment Search Tool (BLAST) analysis against the nonredundant protein sequences database of National Center for Biotechnology Information (NCBI-nr). The results indicate that SK contains the largest species count of 6264, with 129 phyla, followed by SP 5560 species in 44 phyla, RM1 with 5493 species from 128 phyla, RM3 with 4305 species from 131 phyla, SR with 3411 species from 81 phyla, and RM2 3092 species from 100 types of phyla (Table 1). The analysis of the taxonomic community showed that Royal Belum soils are dominated by bacteria (98% in SK, 98% in SP, and 98% in SR), Archaea (0.9% in SK, 0.9% in SP, and 1% in SR), and the remaining were classed under Eukaryota and viruses. Likewise, the soils of Raja Muda Musa are also dominated by bacteria (95% in RM1, 98% in RM2, and 96% in RM3), followed by Archaea (4% in RM1, 1% in RM2, and 3% in RM3), and the remaining were classed under Eukaryota and viruses (Figure 1).

**Figure 2:**
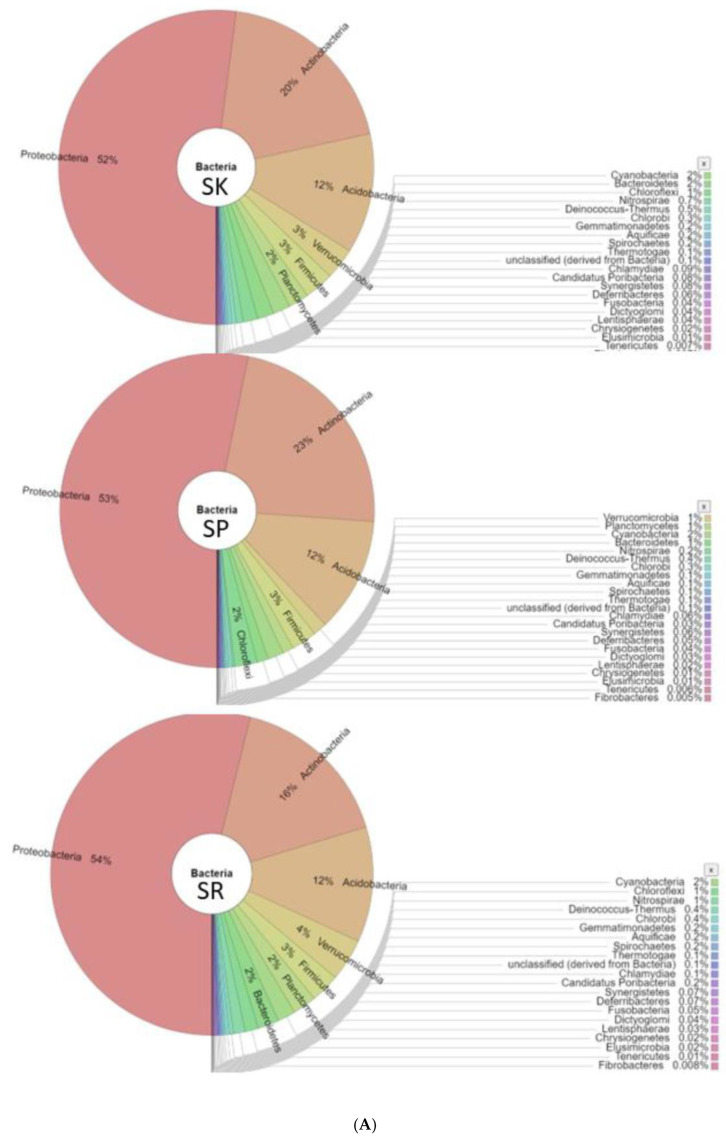

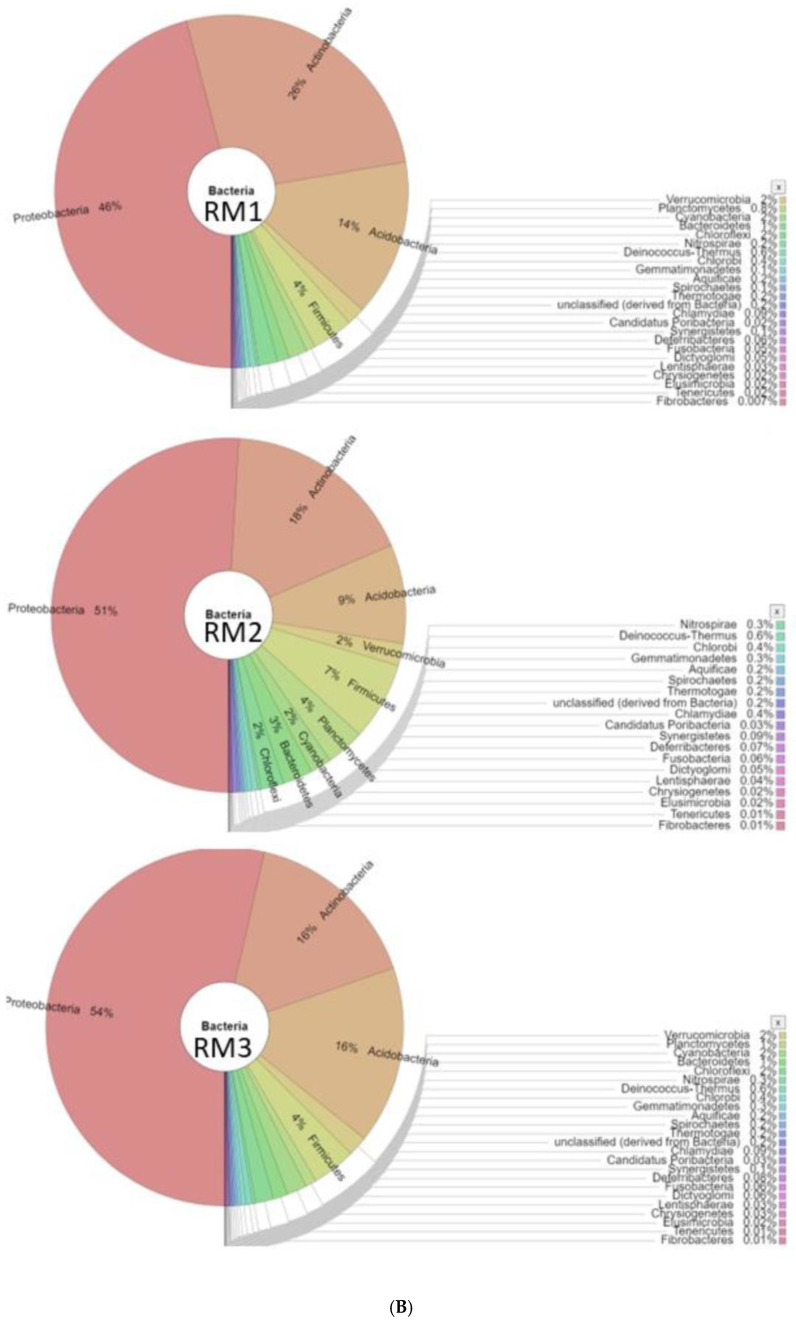
(**A**) Metagenome profiling of total soil bacteriota isolated from RBFR (SK, SP, and SR) based on Krona RSF display. The frequencies of each phylum are presented and reveal the protebacteria as the dominating phylum in all studied sites. (**B**) Metagenome profiling of total soil bacteriota isolated from RMFR (RM1, RM2, and RM3) based on Krona RSF display. The frequencies of each phylum are presented and reveal the *protebacteria* as the dominating phylum in all studied sites.

The bacterial composition of samples from SK, SP, SR, RM1, RM2, and RM3 was further investigated using the MgRast server, where a total of 27 phyla within the bacterial domain were selected among the six samples, namely *Acidobacteria*, *Actinobacteria*, *Aquificae*, *Bacteroidetes*, *Chlamydiae*, *Chlorobi*, *Chloroflexi*, *Chrysiogenetes*, *Cyanobacteria*, *Deferribacteres*, *Deinococcus–Thermus*, *Dictyoglomi*, *Elusimicrobia*, *Fibrobacteres*, *Firmicutes*, *Fusobacteria*, *Gemmatimonadetes*, *Lentisphaerae*, *Nitrospirae*, *Planctomycetes*, *Proteobacteria*, *Spirochaetes*, *Synergistetes*, *Tenericutes*, *Thermotogae*, and *Verrucomicrobia*. The top relative abundances of microbial diversity at the phylum level in percentage for designated sites are presented in Table 2 and Figure 2.

The most dominant sequence is *Proteobacteria*, occurring at 52.15% in RB and 48.28% in RM sequences. Moreover, although *Actinobacteria* is the second-most dominant sequence, it greatly contributes to the Royal Belum and Raja Muda Musa soils, with 19.33% of RB sequences from *Actinobacteria* compared to 19.27% of RM sequences. *Firmicutes* contributed a more than two-fold higher percentage of the total RM sequences than the RB. Conversely, the relative abundance of the phyla *Proteobacteria*, *Nitrospirae*, and *Verrucomicrobia* are significantly higher in SR compared to the other sites. The *Chloroflexi* phylum is relatively abundant in SP. Additionally, *Actinobacteria* is significantly higher in the RM1 sample than others. Firmicutes and Planctomycetes are found in abundance in RM2. *Acidobacteria* and *Gemmatimonadetes* are significantly higher in RM3, while *Deinococcus–Thermus* and *Fibrobacteres* are extremely low in the SP sample. The other remaining phyla do not show any significant difference in gene abundance (Table 2). 

**Figure 3:**
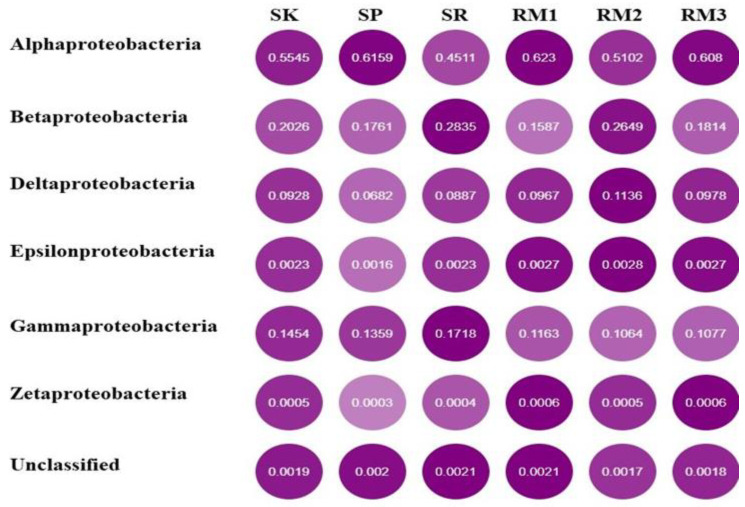
*Proteobacteria* class compositions (abundance matric) of the Royal Belum (SK, SP, and SR) and Raja Muda Musa (RM1, RM2, and RM3) metagenomes as determined by the WGS Shotgun. Taxonomic assignments were performed using MgRast.

Interestingly, differences within the phylum Proteobacteria are observed between the two environments. *Alphaproteobacteria* is relatively more abundant in the RM1 sample with 62.3%, while SR has more *Betaproteobacteria* and *Gammaproteobacteria* (28.35% and 17.18%). RM2 has more *Deltaproteobacteria*, with 11.36%. *Alphaproteobacteria* contributes to a much greater percentage of Proteobacteria in RM (58%) than in RB (54%). Additionally, *Deltaproteobacteria*, *Epsilonproteobacteria*, and *Zetaproteobacteria* are more important in RM than in RB, while *Betaproteobacteria* and *Gammaproteobacteria* are more abundant in RB than in RM (Figure 3). 

### 2.2. Taxonomy of Genes and Bacteria Diversity Involved in Stress Response

**Figure 4:**
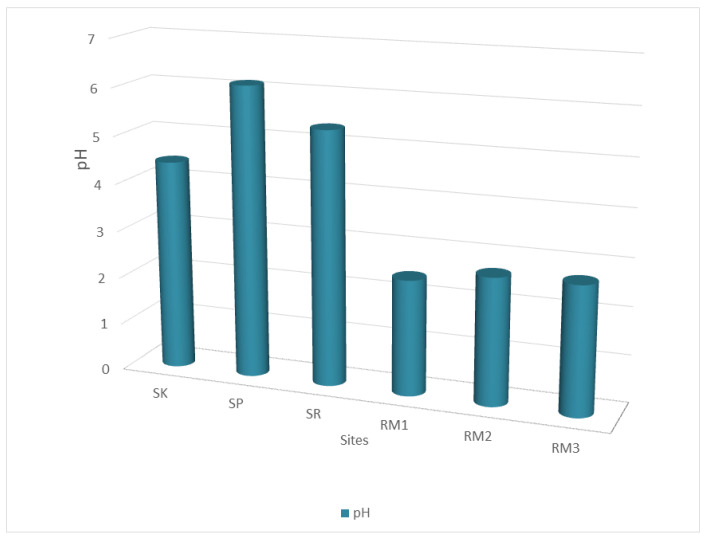
Comparison of pH among samples taken from Royal Belum Reserved Forest and Raja Muda Musa Peat Swamp Reserved Forest.

This study aims to compare the taxonomic diversity of bacteria on two metagenomic profiles of two distinct soil samples from different locations in terms of physicochemical properties and to find bacterial species that respond to abiotic stress conditions. Based on our finding, RMFR soil samples showed higher acidic conditions compared to the soil samples from RBFR. The physicochemical analysis of soils is recorded in Figure 4. The average pH at the Raja Musa Forest Reserve (RMFR) are Raja Musa 1 (RM1, 2.43), Raja Musa 2 (RM2, 2.67), and Raja Musa 3 (RM3, 2.7), indicating that the soil in these areas is acidic compared to the soil at Royal Belum Forest Reserve (RBFR). The pH of soils from the sampling stations in RBFR are Sungai Kooi (SK, 4.42), Sungai Ruak (SR, 5.34), and Sungai Papan (SP, 6.12). A pH between 6.5 and 7.5 is considered optimal for the growth of many plants. RM1 soil exhibits the lowest pH, indicating that soils of the study area are acidic.

**Figure 5:**
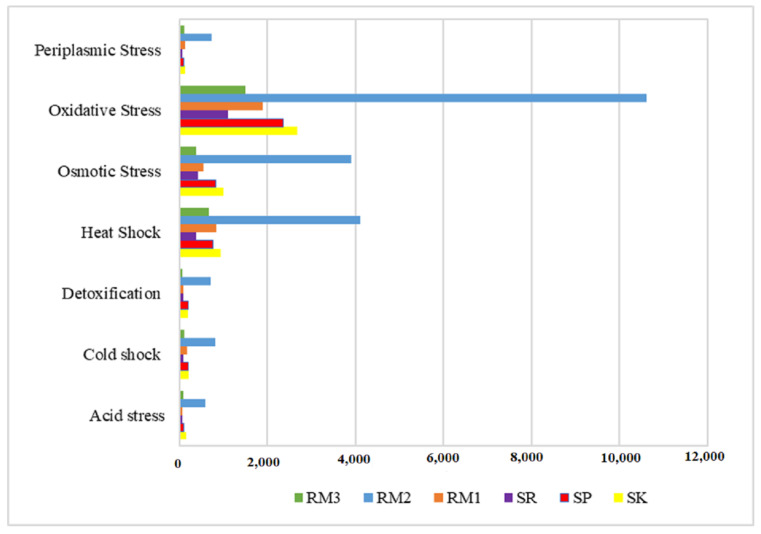
Gene abundances involved in the microbial-degradation pathways of stress response by the WGS Shotgun. Taxonomic assignments for the Royal Belum (SK, SP, and SR) and Raja Muda Musa (RM1, RM2, and RM3) metagenomes were performed using MgRast.

**Figure 6:**
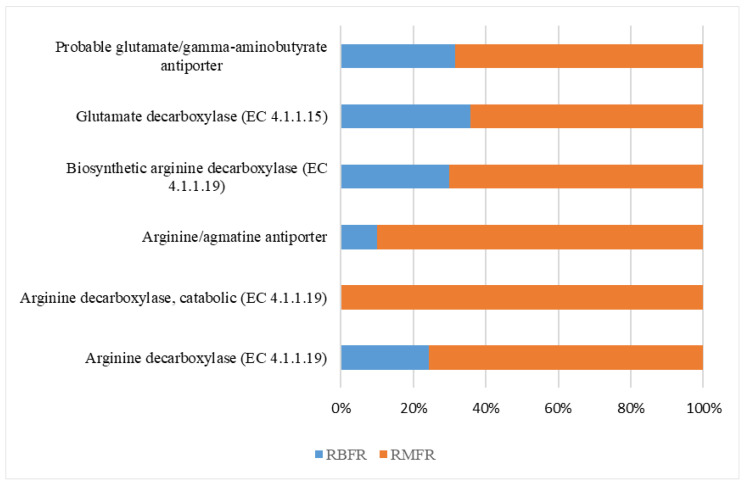
Gene abundances involved in the microbial-degradation pathways of acidic stress-response mechanisms by the WGS Shotgun. Taxonomic assignments for the Royal Belum (SK, SP, and SR) and Raja Muda Musa (RM1, RM2, and RM3) metagenomes were performed using MgRast.

In general, RBFR and RMFR metagenomes contained abundant stress-related genes assigned to various stress-response pathways, many of which did not show any difference between the two sites (Figure 5). The most abundant oxidative stress-related genes occurred among all of the stress-related pathways in each sample, followed by osmotic stress and heat shock. Other genes for stress response detected in this study include phosphate and nitrogen limitations, envelope stress, stringent response, heat and cold shocks, and antioxidant enzymes. Significantly, half of these stress-response pathways in soil metagenomes increased gene abundance with sampling depth, particularly those linked with acidic conditions, as shown in Figure 6.

Metagenomics analysis of soil samples collected from RBFR resulted in 1,391,841 predicted protein-coding regions. Out of this number, 30% (421,409 genes) are classified as functional genes. Approximately 11,751 genes of the functional genes are assigned to stress response. Further breakdown of the stress-response genes reveals 177 genes related to acidic stress. 

In comparison, the detailed MG-RAST analysis of RMFR resulted in 3,586,577 total predicted protein-coding regions. About 30% (1,111,985 genes) are grouped as functional genes. Further analysis of the functional genes has identified 28,750 stress-response genes, with 449 genes related to acidic stress (Table 3).

**Figure 7:**
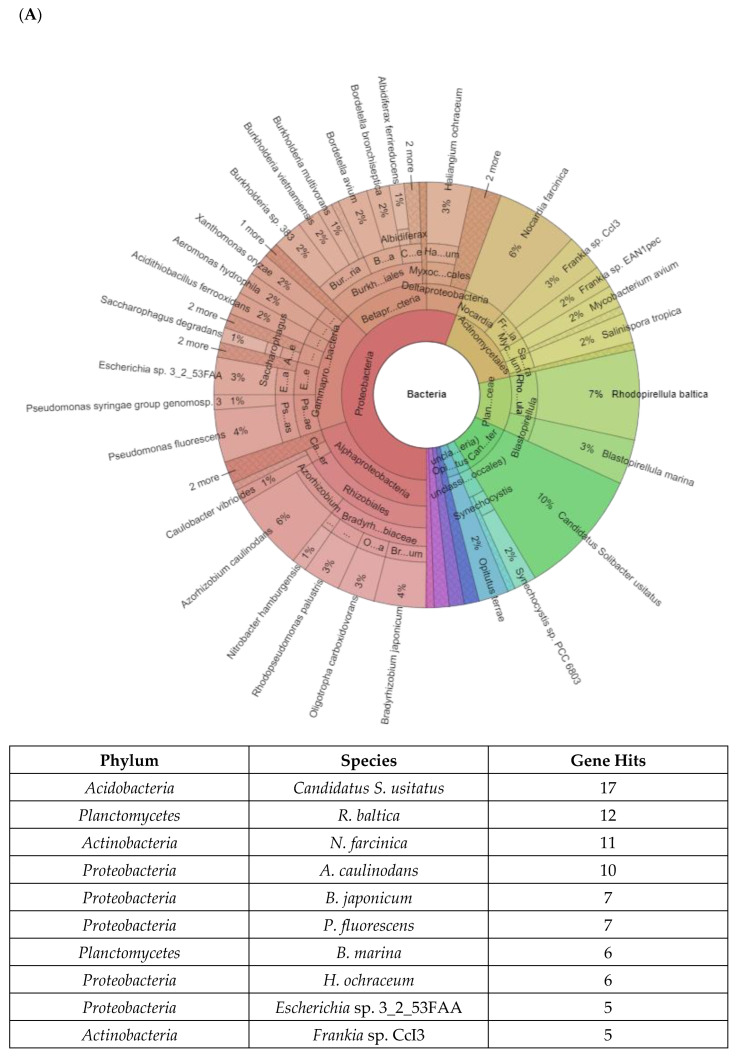

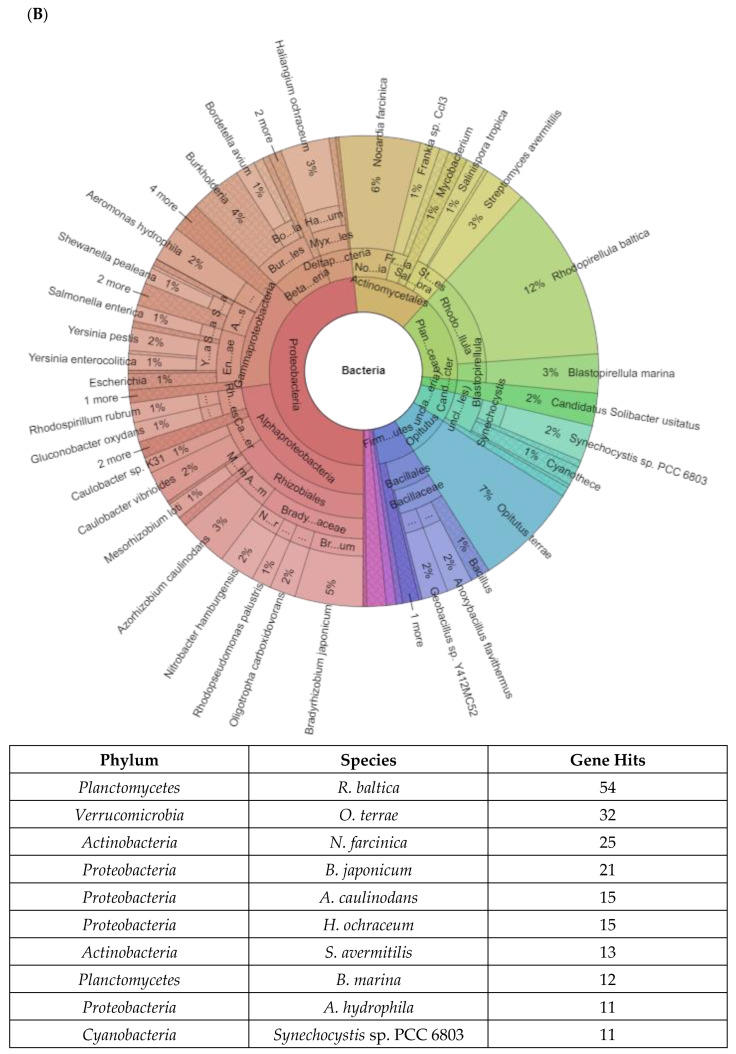
(**A**,**B**) Microbial community compositions of the Royal Belum and Raja Muda Musa soil metagenomes related to acid stress-response genes.

Figure 7A shows the top 10 dominant bacterial species related to acid stress in RBFR, presented in the Krona chart. The most dominant species detected in RBFR, with gene hits as much as 54 times, is *Rhodopirellula baltica* from the *Planctomycetes* phylum. This is followed by *Opitutus terrae* from the phylum *Verrucomicrobia*, with gene hits as much as 32 times. *Nocardia farcinica* from the phylum *Actinobacteria* is detected 25 times. Furthermore, *B. japonicum*, *Azorhizobium caulinodans*, and *Haliangium ochraceum* are bacteria from the *Proteobacteria* phylum, with gene hits as much as 21, 15, and 15. *Actinobacteria* once again dominate the RBFR with the species *Streptomyces avermitilis*, with 13 times gene hits. *Blastopirellula marina*, a species of the *Planctomycetes* phylum, generated 12 times gene hits. Meanwhile, *Aeromonas hydrophila* of the *Proteobacteria* phylum and *Synechocystis* sp. PCC 6803 derived from the *Cyanobacteria* phylum exhibits 11 times gene hits.

Figure 7B shows the top 10 dominant bacterial species related to acid stress in RMFR. *Candidatus S. usitatus* from *Acidobacteria* is the most dominant species, with gene hits as much as 17 times and detected more than *R. baltica*, which is from *Planctomycetes*, with 12 times gene hits. The third and second dominant bacteria do not differ much in gene hit detection, i.e., only one difference in terms of number, and the species are *N. farcinica* derived from *Actinobacteria* phylum. The phylum *Proteobacteria* dominates the RMFR and RBFR soils. The bacteria from the *Proteobacteria* phylum dominate positions of 4, 5, 6, 8, and 9. The species are *A. caulinodans*, *B. japonicum*, *P. fluorescens*, *H. ochraceum*, and *Escherichia* sp. 3_2_53FAA, with gene hits detection as much as 10, 7, 7, 6, and 6 times. *B. marina* of the Planctomycetes phylum dominates the RMFR, with gene hits of six times. The least detection of dominancy in RMFR soil is *Frankia* sp. CcI3 species derived from *Actinobacteria*, with gene hits as much as five times.

## 3. Discussion

Typical dominant bacterial phyla in forest soils are *Acidobacteria*, *Actinobacteria*, *Proteobacteria*, *Bacteroidetes*, and *Firmicutes* [47]. The archaeal community is mostly dominated by the phylum *Thaumarchaeota*, while *Euryarchaeota* and *Crenarchaeota* are less abundant [48].

Forest ecosystems provide a wide habitat for bacteria, including soil and plant tissues and surfaces, streams, and rocks. However, bacteria are especially abundant on the forest floor, soil, and litter. Five phyla, *Acidobacteria*, *Actinobacteria*, *Proteobacteria*, *Bacteroidetes*, and *Firmicutes*, are abundant in most soils. In addition to pH, which seems to be the most important driver of the bacterial community composition in soils, organic matter content, nutrient availability, climate conditions, and biotic interactions affect the composition of bacterial communities. The spatial variation in these parameters is responsible for the presence of hot spots of microbial activity with increased abundance and activity in the soil. 

*Acidobacteria* plays an important role in soil act as “keystone taxa”, acting as the ecosystem engineer. They act as the driver of the bacterial community in terms of structure and functional ecosystem. The phylum is involved in biogeochemical processes, such as C, N, and S cycles [49]. *Actinobacteria* is important in the decomposition of soil, involving the production of carbohydrate-active enzymes (CAZymes) while maintaining the stability of the taxonomic and functional composition of the soil. It is also enhanced by N fixation [50]. CAZymes are also produced by *Bacteroidetes* for survival in soil, where the enzymes target the abundant glycans in the soil [51]. 

A dominant phylum found in the RBFR but not in the RMFR is *Cyanobacteria*. The *Cyanobacteria* phylum is involved in the nutrient exchange, including vitamins, and grows well at high temperatures. *Cyanobacteria* proliferation is influenced by P and N [52]. A study on the soil microbial community reported that the dominance of Proteobacteria in soil correlated with carbon-rich soil [53], as the *Proteobacteria* is directly involved in the C cycle of the soil. In comparison, the phylum Firmicutes and its order *Clostridiales* act as the decomposer in soil. The phylum is found to be abundant in compost soil, and it also greatly impacts the conversion of organic matter [54].

Analysis of the long-read data also showed thousands of organisms with an abundance of <0.1% in all samples. All of these species are classified as minorities from the overall diversity of bacteria found in the study area. Stress-response coding genes are significantly more abundant than rare genomes, indicating that rare species can affect the pH turnover capability and provide resilience to changing environmental conditions. Overall, the analysis showed that the diversity of closely related strains and unusual species is a significant part of the population. According to Penn et al. (2019) [55], the pH of soil affects chemical solubilities by influencing the ionization degree. It is noteworthy that the pH values at the two sites are integrated results due to numerous interactions between the cations and anions in the soil solution [56]. The large difference in pH at the two sites implies the distinct geochemical environment of both sites. It can be presumed that pH played a definite role in the diversity and composition of the bacterial community [57,58]. Pietri et al. (2008) revealed statistically significant relationships between soil pH and biomass C (R2 = 0.80, *p* < 0.001), biomass ninhydrin-N (R2 = 0.90, *p* < 0.001), organic C (R2 = 0.83, *p* < 0.001), and total N (R2 = 0.83, *p* < 0.001), confirming the importance of soil organic matter and pH in stimulating microbial biomass growth [59]. 

The findings of this study revealed seven stress responses, i.e., periplasmic stress, oxidative stress, osmotic stress, heat shock, detoxification, cold shock, and acid stress from the six sampling sites, i.e., RM1, RM2, RM3, RB1, RB2, and RB3. Most of the stress-response gene counts, particularly heat and osmotic stress, are found highest in RM2. These stress-response genes included heat and osmotic stress. 

The soil samples used for this study are classified as acidic. Hence, the attention is given to reveal the abundance of acid stress-response genes in the soil sample. Six genes are found involved in acid stress, which are probably glutamate/gamma-aminobutyrate antiporter, glutamate decarboxylase (EC4.1.1.15), biosynthetic arginine decarboxylase (EC 4.1.1.19), arginine/agmatine antiporter, arginine decarboxylase EC 4.1.1.19), and arginine decarboxylase (EC 4.1.1.19). The probable glutamate/gamma-aminobutyrate antiporter gene, biosynthetic arginine decarboxylase (EC 4.1.1.19), and arginine decarboxylase (EC 4.1.1.19) are 70% higher in RMFR than RBFR. Similarly, it has more than 60% of the glutamate decarboxylase (EC4.1.1.15) gene and more than 90% of the arginine/agmatine antiporter gene than RBFR. Interestingly, the arginine decarboxylase (EC 4.1.1.19) gene is only present in the RMFR soil and none is found in the RBFR soil sample.

## 4. Materials and Methods

### 4.1. Experimental Sites

**Figure 8:**
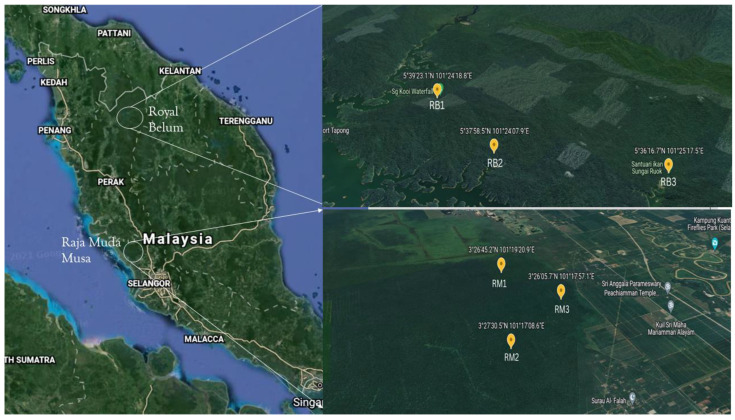
The coordinates and elevation for Royal Belum Reserved Forest sampling sites SK, SP, and SR, and Raja Muda Musa Peat Swamp Reserved Forest sampling sites RM 1, RM2, and RM 3.

The RBFR is situated in the northern part of Malaysia in the Perak State, where soils were taken in May 2018. This forest is the largest continuous forest complex in Peninsular Malaysia, covering an area of 117,500 hectares of thick forest stretching into the Thailand–Malaysia border. The area has high rainfall of approximately 2560 mm/year, with average temperatures during the collection time at 28–30 °C and pH of the samples between 4.42 and 6.12. The RMFR situated in North Selangor Peat Swamp Forest is a remnant of a larger peat swamp forest that has been reduced by drainage, conversion to agriculture, and logging. The climate is tropical with a mean annual rainfall of more than 200 cm per year and an average temperature of 28 °C. The peat substrate is several meters deep, lying on a bed of marine alluvial clay, and is perpetually waterlogged with the forest floor becoming submerged during rainy periods. The water is acidic (pH 3–4), has a characteristic “blackwater” color due to high concentrations of tannins and humic acids. About 54 soil samples were collected from 6 distinct locations with 9 replicates of soil sample per site (1 kg each). The soils were randomly collected from top surface sediments to a depth of 5 cm. The samples that contained mainly soil and plant biomass residues were mixed to represent one site and used for all experiments in this study. Samples were stored at −80 °C until DNA extraction and preserved accordingly to maintain both quality and accuracy of the metagenomics data. Site A (RB) soil sample was collected at Sungai Kooi (SK), while site B (RB) was located at Sungai Papan (SP), and site C (RB) was located at Sungai Ruok (SR). For RMFR, the soil sample was collected randomly in the peat swamp reserved forest using the three-point sampling method. For cumulate soil samples in the forest, the sample location was selected by implementing the random pattern protocol. The major coordinates and elevations for both sites are presented in Figure 8.

### 4.2. Soil Bacterial Metagenome Extraction

The DNA samples were extracted within 24 h after sample collection according to the modified procedure elaborated for soil material described by [60] using the DNeasy PowerSoil Kit. The DNA samples were subjected to three quality control (QC) procedures using a Nanodrop spectrophotometer (Thermo Fisher, DE, USA) at A260/280 UV length, gel electrophoresis profiling, and Qubit fluorometric quantitation using fluorescent dye, to ensure the intactness of the samples [61]. DNA samples that passed the QC were purified using magnetic beads and agent court to remove tannin and undergo a mechanical shearing procedure using a Covaris Sonicator (Woburn, MA, USA) to ensure a standardized DNA sample length between 300 and 350 bp measured by an Agilent Bioanalyzer 2100 (Santa Clara, CA, USA) [62,63,64]. The samples were measured by Qubits to ensure that the total recovery after purification was more than 70%, and the total DNA per 50 µL volume was 1 µg before adenylation of the 3′ end. Individual barcodes of index adapter sequences were added to each DNA fragment during library preparation so that each read could be identified and sorted before the final data analysis for the flow cell chip (Table S1). The samples were sequenced using the Illumina NGS (Illumina Next Generation Sequencing) paired-end technology [65], and the reads were aligned and went through a preprocessing phase in the bioinformatics pipeline that included alignments and quality trimming with Solexa QA++ (Hayward, CA, USA) [66], contigs assembly with metaSPAdes [67], and analysis using MEGAN6 (Metagenome Analyzer) [68] and MgRast (http://metagenomics.anl.gov, accessed on 24 March 2021) [69] to determine the genetic repertoire of the microbiome.

### 4.3. Gene Annotation and Sequence Analysis

The raw sequence quality was checked, and the reads were trimmed accordingly using FastQC (Version 0.11.5 released), a tool provided by the Babraham Institute (Cambridge, UK), which simplifies the QC of high-throughput sequencing pipelines. Solexa QA++ was used in the Command Line Interface [70] of the DynamicTrim application, where the sequences were trimmed based on Qphred < 20 and the LengthSort command sequences shorter than 50 bp were removed. The cleaned sequences were paired and shuffled to produce high-quality sequences before being assembled using the metaSPAdes (version 3.13.0). The high-quality sequences were mapped to the final assembly, and the coverage information was generated through the default parameters of the bbmap (Version 38.25), except for ambiguous = random. Diamond software was used to BLAST the predicted genes against the nonredundant protein sequences database of NCBI (https://www.ncbi.nlm.nih.gov/ accessed on 24 March 2021) with default settings [71] using BLASTP (best hit with E < 0.001). Functional annotation was conducted by aligning sequencing reads against the KEGG database (Release 84.1) [72] using the MEGAN6 software (Version 6.11.7; Huson, 2016) with the parameter setting of BLASTP [71] according to the LCA algorithm. Finally, the gene read numbers for each sample were normalized based on the median read number. The relative abundances (percentage) of genes were calculated related to the annotated reads and used for subsequent analyses. The M5 nonredundant protein database (M5NR) was used for taxonomic annotation and the SEED and Clusters of Orthologous Groups databases for functional annotation. The best BLASTx hit was used to identify the sequences with a minimum alignment length of 15 bp and an e-value cutoff of e < 1 × 10^−5^ and a 95% confidence interval. Functional annotation of the most abundant taxa was performed using the filter option. The same was carried out for a selected group of genes to reveal the responsible taxa. The shotgun metagenomics sequence data used in this study were deposited in the MG-RAST server [69] under project ID mgp94971 and mgp94737.

## 5. Conclusions

Through our study, we investigate the microbial diversity in two distinct ecosystems varied by the acidity of the soils. These very different ecosystems became an abiotic factor where the bacterial communities in these two areas have evolved symbiotically and complement each other to form a stable soil ecosystem. This study reveals that the rainforest and peat swamp soils harbor distinct microbial populations and metabolic processes dominated by Proteobacteria. Even though the same detected phyla are shared among the studied sites, their abundances are different and correlated with the specific soil. Our study has also discovered through KEGG pathway analysis the genes involved in the acid stress-response regulation, which has led to the identification of the responsible genes, which are probably glutamate antiporter, glutamate decarboxylase, biosynthetic arginine decarboxylase, arginine/agmatine antiporter, and arginine decarboxylate. Thus, our study suggests that the pH of the soil has significant effects on the taxonomic, gene, and metabolic diversity. The insights into the functional structuring of the microbiomes gained with this study provide a basis for understanding the processes contributing to ecosystem services. 

## Figures and Tables

**Table 1:** Blast hits against the nonredundant protein sequences database of NCBI (NCBI-nr).

Forest Types	Sites	Phylum	Species
Rainforest	SK	129	6264
	SP	44	5560
	SR	81	3411
Peat Swamp Forest	RM1	128	5493
	RM2	100	3092
	RM3	131	4305

**Table 2:** Top relative abundances of microbial diversity at phylum level in percentage for three sampling sites in Royal Belum Reserved Forest and Raja Muda Musa Peat Swamp Reserved Forest that hit with the NCBI-nr database. The amount of hits refers to the abundances of a specific phylum in the population.

Phylum	Royal Belum Bacteria Abundances (%)				Raja Muda Musa Peat Swamp Bacteria Abundances (%)			
	SK	SP	SR	Total	RM1	RM2	RM3	Total
*Proteobacteria*	51.18	52.38	52.89	52.15	43.66	49.68	51.51	48.28
*Actinobacteria*	19.21	22.6	16.19	19.33	2.17	17.16	15.48	19.27
*Acidobacteria*	12.16	11.6	11.34	11.7	13.68	8.83	15.42	12.64
*Verrucomicrobia*	3.17	1.4	4.19	2.92	1.57	1.98	1.73	1.76
*Firmicutes*	2.97	2.6	2.86	2.81	3.6	6.98	3.79	4.79
*Bacteroidetes*	1.68	1.15	2.09	1.64	1.03	2.67	1.27	1.66
*Planctomycetes*	2.05	1.11	2.34	1.83	0.79	3.42	0.97	1.73
*Cyanobacteria*	1.75	1.59	1.78	1.71	1.63	1.91	1.72	1.75
*Chloroflexi*	1.47	2.29	1.3	1.69	1.65	2.02	1.58	1.75
*Nitrospirae*	0.67	0.2	1.14	0.67	0.23	0.26	0.27	0.25
*Deinococcus–Thermus*	0.46	0.04	0.42	0.31	0.56	0.56	0.54	0.55
*Others*	3.23	3.04	3.46	3.24	6.43	4.53	5.72	5.57

**Table 3:** Total of protein-coding region to functional, stress-response, and acidic stress-response genes.

Total	Protein-Coding Region	Functional Genes	Stress-Response Genes	Acidic Stress-Response Genes
Royal Belum	1,391,841	421,409	11,751	177
Raja Musa	3,586,577	1,111,985	28,750	449

## Data Availability

Not applicable.

## Supplementary Materials

The following supporting information can be downloaded at: https://www.mdpi.com/article/10.3390/ijms24010872/s1.
