# Supplementary material for: Topographically Distinguished Microbiome Taxonomy and Stress-Response Genes of Royal Belum Rainforest and Raja Muda Musa Peat Swamp Revealed through Metagenomic Inquisition
Source: Int J Mol Sci. 2023 Jan 3;24(1):872. doi: 10.3390/ijms24010872 (PMC9821613; doi:10.3390/ijms24010872)
Supplement: Supplementary file 1 [file ijms-24-00872-s001.zip › ijms-2071322-supplementary.pdf]

S1: DNA Quantity of Royal Belum and Raja Musa sites

Sample Name	Sample ID	Qubit Concentration (ng/µL)	Molarity Estimation (nm)	Size (bp)
Royal Belum 1	SK	10.94	27.858	595
Royal Belum 2	SP	7.84	21.288	558
Royal Belum 3	SR	5.00	13.528	560
Raja Musa 1	RM1	9.87	25.114	573
Raja Musa 2	RM2	12.05	29.719	583
Raja Musa 3	RM3	8.92	23.528	584
